# Does Spatial Navigation Have a Blind-Spot? Visiocentrism Is Not Enough to Explain the Navigational Behavior Comprehensively

**DOI:** 10.3389/fnbeh.2017.00154

**Published:** 2017-08-18

**Authors:** Mateusz Hohol, Bartosz Baran, Michał Krzyżowski, Jacek Francikowski

**Affiliations:** ^1^Department of Logic and Cognitive Science, Institute of Philosophy and Sociology, Polish Academy of Sciences Warsaw, Poland; ^2^Copernicus Center for Interdisciplinary Studies Kraków, Poland; ^3^Department of Animal Physiology and Ecotoxicology, Faculty of Biology and Environmental Protection, University of Silesia Katowice, Poland

**Keywords:** spatial navigation, geometric module, view-matching, insect studies, multimodal integration

A few years ago Wystrach and Graham (2012b) asked: “What can we learn from studies of insect navigation?” They identified that complex navigational behavior of insects can be explained via fairly simple mechanisms, such as view-matching (Wehner and Räber, 1979; Cartwright and Collett, 1983), without referring to the high-level mental mechanisms (Cheng, 1986). Furthermore, they suggested that since the navigational behavior of vertebrates, show significant convergence with insects' behavior, it is justified to consider mechanism based on egocentric views before assuming the existence of higher-level mechanisms.

Such a point of view seems to fall in line with a broader consensus, and wider paradigm shift that has taken place in recent years. For instance, Cheng (2008), the former proponent of the high-level geometric module, suggested that view-matching models of insects' navigation, can be directly implemented in the studies on the spatial navigation of vertebrates, and even human. This paradigm-shift was also driven by successes in implementing view-matching to behavioral robotics (Möller and Vardy, 2006).

To test for the use of egocentric view (i.e., view based matching hypotheses), most studies use pixel-by-pixel image comparison, and some results in both vertebrates and insects reject the prediction made by such image comparison (Lehrer et al., 1988; Lee et al., 2012). However, it is argued that rejecting pixel-by-pixel comparison is not rejecting the use of view-based matching mechanisms, as animal views are not “images” but can encode parameters such as depths, motion, edges or specific features, which varies across species (Wystrach and Graham, 2012a). In this light, pixel-by-pixel models of insect navigation seem unrealistic and should not be understood literally, but rather as a proxy for quantifying surroundings familiarity. However, and beyond that debate, it is clear that reorientation can be accomplished in ways excluding any type of view-based-matching mechanism, such as when accomplished by a blindfolded human (Sturz et al., 2013). Furthermore, Cheng et al. (2013) pointed out that in the contemporary literature on spatial navigation there are several competing approaches, and the field lacks a unified research paradigm.

In this paper, we argue that the issues described arise not because of the lack of theoretical inspiration, but rather due to an insufficient understanding of the subtleties of insect behavior. In our view, implementation of the insects' models of navigation in the explanation of the vertebrates' spatial behavior omits some important aspects, i.e., multimodal integration. Thus, we want to ask again the initial question posed by Wystrach and Graham (2012b) pointing out that significant progress in insects' research, which suggests that we might have had underestimated insects' cognitive abilities (Loukola et al., 2017; Peng and Chittka, 2017). Those results demonstrated insects' capacity to obtain abstract information from multimodal input during complex tasks. Movement through a real environment provides a variety of cues, not only visual ones, thus in the following article we argue that multimodal integration is crucial to navigation.

## Visiocentrism in the studies on spatial navigation

Vertebrates' capacity for spatial navigation has been traditionally perceived as the product of the activity of the so-called “geometric module” (Fodor, 1983), which encodes only the geometric shape of the environment via purely visual input (Gallistel, 1990). This thesis was derived primarily from the results of behavioral studies (Cheng, 1986), in which familiarized animals after a disorientation phase, searched for a reward hidden in a given corner of a rectangular arena, relying mainly on the visually perceived geometry of the apparatus (see Thinus-Blanc et al., 2010 for review). The geometric module was thought to be localized in the hippocampus (Vargas et al., 2004) and separated from both domain-general processes as well as other domain-specific cognitive modules (Gallistel, 1990).

The further studies conducted with various species, especially human (Hermer and Spelke, 1994) and non-human primates (Gouteux et al., 2001), have showed that vertebrates' spatial navigation sometimes depends on the productive combination of geometric and non-geometric information. Namely animals can optimize task performance by using recognized featural cues or landmarks. These results have triggered conceptual shift. According to Spelke et al. (2010), spatial navigation depends on two “core cognitive systems” for processing three-dimensional spatial layouts and two-dimensional visual forms respectively.

Spatial navigation is, however, still thought to be a product of high-level mechanisms which process information retrieved from visual input. Even earlier attempts (Cheung et al., 2008) to implement a view-matching approach (Wehner and Räber, 1979; Wehner, 2003) to vertebrate's spatial navigation studies haven't changed the research perspective as radically as it seemed. The tendency to focus on solely visual-driven mechanisms in studies of spatial navigation, with simultaneous neglect of other modalities, we have decided to refer to as: *visiocentrism*. It seems that the visiocentric perspective still dominates the study of insect and vertebrate navigation.

In the following sections, we aim to challenge this viewpoint from evolutionary, neurophysiological and behavioral perspectives. We point out that the current state of understanding of the evolutionary economics seems incoherent with visiocentric models of navigation, in terms of insect nervous systems and information processing. Furthermore, we discuss evidence from behavioral studies utilizing non-visual/mixed approaches, to support our claim that multimodal integration plays crucial role in spatial navigation.

## Spatial navigation in little brains

During phylogenesis, nervous systems tend to undergo specialization in order to achieve fitness to the ecological niche of a particular species (Liebeskind et al., 2016). Despite divergence of the evolutionary pathways of arthropods and vertebrates, many authors point out functional and structural similarities in their visual systems, specifically in neural circuits specialized for visual processing (Borst and Euler, 2011; Shih et al., 2015). The most important feature of all these systems is an economy of neuronal wiring (Chen et al., 2006). This principle is observed on retina level (neuronal convergence) and in the central nervous system (Laughlin et al., 1998). Basic restrictions of nervous system complexity are the size of the head cavity, and high energy demand, as neuronal conduction of information on the axonal and synaptic level is very energy-intensive (Laughlin et al., 1998). By understanding the neuronal economy, the functional economy can be postulated. The amount of information which can be transmitted through the nervous system in the unit of time is restricted by many factors (Bullmore and Sporns, 2012). The solution to this problem is to, at very early stage, select, reduce and compress information (Nityananda, 2016).

The second important mechanism used to deal with excess information in the nervous system, is the set of models and algorithms (Wehner, 1987; Webb, 2004). This clearly demonstrates that authentic reproduction of the external environment is impossible, and brains create a simplified representation of an environment with emphasis on key elements (Doyle and Csete, 2011). Visual information on the level of optical lobes is not analyzed as a unitary structure but is divided on movement reception, related to optomotor response, and object features such as color, small object movement, and shape (Dunbier et al., 2012). We know that, in insects, sensory information is analyzed in small loops specialized in the preliminary analysis, known as the small-world network. Subsequently, information flow is redirected to main associative structures: mushroom bodies (MBs) and central complex (CC) (Strausfeld et al., 1998). It is uncommon for these structures be connected directly by afferent or efferent pathways, whether sensory or effectory. Additionally, visual information does not remain separate but is coupled with other modalities (Borst and Euler, 2011). The MBs and CC are structures with numerous outputs and inputs of various modalities, and thus are considered as a higher-order multimodal information integration centers (Wessnitzer and Webb, 2006; Avargues-Weber and Giurfa, 2013; Giurfa, 2013).

Depending on evolutionary pressures, neuronal background can differ between insect species (Wehner, 1987). Nevertheless, presence of MBs and CB is universal in *insecta* class and, as it was stated above, those structures play crucial role in spatial memory and spatial navigation. While it seems counterintuitive that units described as sensory afferents can carry more than one modality, intracellular recordings have revealed that many afferents to both structures (MBs and CC) are multimodal (Li and Strausfeld, 1997). This suggests that higher processing instances does not have access to raw sensory information, and locomotion cannot be regulated directly by simple activity of retina cells. Additionally, during transmission, visual information from eyes undergoes intensive processing (Zipursky and Sanes, 2010). In all insects, single neurons connecting optic lobes with higher associative structures can be observed. This feature can be seen as a manifestation of economical wiring. These neurons originate in areas that receive collaterals and terminals of visual interneurons, from the ipsilateral and contralateral optic lobes, providing consistent information flow (Li and Strausfeld, 1997; Mizunami et al., 1998). Connections between small local loops are usually weaker (contain fewer neurons) than internal connections. It can be an advantage for noise-limited signaling systems. A low capacity pathway (fewer neurons) transmits information in a more economically efficient way, providing better distribution of information among different pathways (Laughlin et al., 1998). The main function of nervous systems is extract and track environmental regularities. Knowledge of these regularities is utilized to anticipate the consequences of movements, thus allowing the most adaptive action selection (Webb, 2012). Those regularities span, however, across various modalities, which appears to be reflected in organization of insects' brains. Therefore, we claim that the visiocentric approach neglects crucial aspects of insects' brain anatomy.

## Beyond visiocentrism

In recent decades, we can observe a change in the approach to insect navigation research. The vision-based strategies, especially the view-matching, has been gaining more and more attention (Wystrach and Graham, 2012a; Avargues-Weber and Giurfa, 2013). The discovery of mammalian-like rotational errors of ants navigating in rectangular arenas (Wystrach and Beugnon, 2009) and the success of view based matching in explaining these results (Stürzl et al., 2008; Wystrach et al., 2011) led to the dissemination of these view-matching models in the vertebrate literature, and, as a corollary, strengthened the unjustified perception of insects as rather simplistic, yet highly adapted agents.

The described shift caused the emergence of visiocentrism, however current data on a neuronal foundation of navigational processes strongly suggest that this approach may be incomplete. According to the arguments already presented and to recently developed models (Webb and Wystrach, 2016; Roper et al., 2017), the insect's brain possesses architecture adapted for sensory integration rather than processing single modalities separately. This data is coherent with observations concerning the use of non-visual or combined cues in insects (Chittka et al., 1999; Thiélin-Bescond and Beugnon, 2005; Wystrach and Schwarz, 2013; Buehlmann et al., 2015; Minoura et al., 2016; Raderschall et al., 2016).

Gallistel (1990) argued that the geometric module is evolutionarily justified, since geometric information remains stable in contrary to changes of the features. A similar argument may be formulated for multimodal integration. When various modalities are considered, chances of misrecognition of object or place are much lower. Even if single modality featural cues change, chances are that in other modalities features may remain: food will retain its smell and taste even if it is smashed or cut. The visual system is one of the most energetically demanding and structurally elaborated systems in the brain (Wong-Riley, 2010), therefore managing computationally complex tasks using only visual instances of the brain seems evolutionary uneconomical. Visual information may be supplemented by other senses to provide more reliable information about surroundings, simultaneously reducing metabolic cost.

Likewise, vertebrates' hippocampi, like the CB and MBs of insects, are provided with inputs from all sensory modalities (Ravassard et al., 2013). These structures are known to be crucial in navigational processes (Mizunami et al., 1998; Pfeiffer and Homberg, 2014), and therefore it is hardly a surprise that senses other than vision are utilized during navigation. Even without vision, blind rats could successfully accomplish navigation if place cells were functioning correctly (Save et al., 1998). In the case of mammalian navigation, there is direct evidence of multimodal input role in the function of place cells (Ravassard et al., 2013). Although, what is surprising, is that the studies investigating reorientation almost entirely neglect this aspect (Cheng, 2008) despite the known role of multimodal integration in perception (Cheng et al., 2007). Therefore, we claim that it would be beneficial for studies on spatial navigation, both in invertebrates and vertebrates, to expand perspective further and, include non-visual modalities. This perspective may be of great value, especially when questions considering the internal representation of space are asked.

## Author contributions

MH, BB, MK, and JF reviewed the literature and developed the theoretical stance. MH, BB, MK, and JF wrote the manuscript. MH, BB, MK, and JF reviewed and accepted its final version.

### Conflict of interest statement

The authors declare that the research was conducted in the absence of any commercial or financial relationships that could be construed as a potential conflict of interest.
